# Level of adherence to prescribed exercise in spondyloarthritis and factors affecting this adherence: a systematic review

**DOI:** 10.1007/s00296-018-4225-8

**Published:** 2019-01-16

**Authors:** M. T. McDonald, S. Siebert, E. H. Coulter, D. A. McDonald, L. Paul

**Affiliations:** 1 Rheumatology Service, NHS Greater Glasgow and Clyde, Glasgow, Scotland, UK; 2 School of Health and Life Sciences, Glasgow Caledonian University, Glasgow, Scotland, UK; 3 Institute of Infection, Immunity and Inflammation, University of Glasgow, Glasgow, Scotland, UK; 4 Nursing Midwife and Allied Health Professional Research Unit, Glasgow Caledonian University, Glasgow, Scotland, UK

**Keywords:** Spondyloarthritis, Adherence, Exercise, Physiotherapy

## Abstract

Adherence is a primary determinant of the effectiveness of any intervention. Exercise is considered essential in the management of spondyloarthritis (SpA); however, the overall adherence to exercise programmes and factors affecting adherence are unknown. The aim of this systematic review was to examine measures of, and factors influencing adherence to, prescribed exercise programmes in people with SpA. A search was performed in August 2018 using five data bases; the Cochrane library, CINAHL, EMBASE, MEDLINE, and Web of Science Collections. Inclusion criteria were: studies with adults (> 18 years) with SpA, with a prescribed exercise intervention or educational programme with the aim of increasing exercise participation. Article quality was independently assessed by two assessors. Extracted descriptive data included: populations, interventions, measures of adherence and factors affecting adherence. Percentage adherence rates to prescribed exercises were calculated if not reported. Nine studies were included with a total of 658 participants, 95% of participants had a diagnosis of ankylosing spondylitis. Interventions and measurement of adherence varied, making comparisons difficult. Rates of adherence ranged from 51.4 to 95%. Single studies identified; adherence improved following educational programmes, and higher disease severity and longer diagnostic delays were associated with higher adherence. Conflicting evidence was found as to whether supervision of exercise improved adherence. Three consecutive studies demonstrated adherence reduced over time. Adherence to prescribed exercise in SpA was poorly reported and predominately for people with AS. The levels of adherence and factors affecting prescribed exercise in SpA remain unclear. Future research should measure adherence across a longer time period and investigate possible factors which may influence adherence.

## Introduction

Spondyloarthritis (SpA) describes a group of inter-related inflammatory arthritis with a prevalence of 0.4–2.4% and an incidence rate of 1–16.4/100,000 in Europe [1]. SpA subsets include ankylosing spondylitis (AS), non-radiographic axial SpA, reactive arthritis (reA), enteropathic arthritis, psoriatic arthritis (PsA) and historically undifferentiated spondyloarthropathy (uSpA) [2, 3]. These conditions share common genetic, pathophysiological and clinical features [2, 4]. AS is the prototypic form of axial SpA which typically starts in the second or third decade of life [5].

Exercise is essential in the management of SpA to maintain or improve mobility, strength, cardiovascular health, function, quality of life and to limit spinal deformity [3]. Most literature studying exercises in SpA have used AS populations and predate the ASAS classification criteria [6], so generalising to SpA as a whole should be done with caution [7]. Evidence shows that exercise improves AS clinical outcomes [8] with guidelines stating that people with AS should exercise frequently at every stage of their condition [9]. Exercise may have a role in attenuating a systemic anti-inflammatory response. This has not yet been proven in the SpA population; however, Level 1 evidence supports that exercise improves disease activity in AS [9].

Adherence refers to the extent to which a person’s behaviour corresponds with the recommendations from a healthcare provider [10]. The term adherence is preferable to the more traditional term of compliance which implies that healthcare providers give instructions which patients passively follow [11]. The term concordance is increasingly used and refers to the consultation process between healthcare provider and patient [12, 13]. However, it cannot be easily measured, and so adherence is preferred within quantitative studies. When considering prescribed exercise programmes; adherence can relate to whether people undertake the prescribed number of exercise sessions and/or; the number of exercises during each completed session, the intensity of exercise within each session or time taken to complete the exercise session [14].

Adherence to exercise programmes appears to be central to the therapeutic success of exercise, with research in people with osteoarthritis indicating adherent patients have better outcomes [15]. Non-adherence to prescribed exercise can reach 70% within other patient populations [16, 17] but the extent is not known within SpA. Exercise programmes in AS should be prescribed based on assessment findings and aim for a high frequency, e.g. five times per week [9, 18]. Adhering to these guidelines is likely to be challenging for both people with SpA and clinical/exercise professionals supporting them, and it is possible adherence may be lower than in other clinical conditions.

Adherence to exercise programmes may be influenced by multiple personal and interventional factors [10]. These factors have been studied in other patient populations [14, 16, 19–23]. Low self-efficacy, depression and pain were associated with reduced adherence [14, 19, 22]. The type and mode of delivery of exercise interventions such as supervised exercise sessions, goal setting and patient education have been shown to increase adherence [17, 21, 22, 24]. The factors which influence adherence to exercise in SpA have not been reviewed. The characteristics of SpA differ from other conditions and thus so might the factors which influence exercise adherence.

The aim of this systematic review was therefore to examine the rates of adherence to prescribed exercise and the factors reported to influence adherence in people with SpA.

## Methods

### Search strategy

The present systematic review follows the preferred reporting items for systematic reviews and meta-analyses (PRISMA) guidelines [25]. A search was performed in August 2018 using five databases: the Cochrane library, CINAHL (1982–March 2018), EMBASE (1989–March 2018), MEDLINE and Web of Science Collections. The search included specific keywords and combined Medical Search History (MeSH) headings were explored for greater depth (Table 1). Date of publication was not restricted. Reference lists of relevant articles were also hand searched.

**Table 1:** Keywords relating to search

1.	Enteropathic arthritis
2.	Reactive arthritis
3.	Seronegative spondyloarthritis
4.	Ankylosing spondylitis
5.	Axial spondyloarthritis
6.	Spondyloarthritis
7.	Psoriatic arthritis
8.	1 OR 2 OR 3 OR 4 OR 5 OR 6 OR 7
9.	Exercise
10.	Muscle strength
11.	Flexibility exercise
12.	Physical therapy modalities
13.	Exercise therapy
14.	Physical activity
15.	Resistance training
16.	Physical fitness
17.	Sport
18.	Movement therapy
19.	Stretching
20.	Educational programme
21.	Walking
22.	Yoga
23.	Hydrotherapy
24.	5 OR 6 OR 7 OR 8 OR 9 OR 10 OR 11 OR 12 OR 13 OR 14 OR 15 OR 16 OR 17 OR 18 OR 19 OR 20 OR 21 OR 22 OR 23
25.	Adherence OR patient adherence OR guideline adherence
26.	Concordance OR patient concordance OR guideline concordance
27.	Compliance OR patient compliance OR guideline compliance
28.	24 OR 25 OR 26
29.	27 AND 23 AND 7

### Inclusion/exclusion criteria

Articles were included if the participants were over 18 years old and had SpA, including AS, non-radiographic axial SpA, ReA, PsA, uSPA or enteropathic arthritis, or if the study had a mixed population but the data related to the SpA population could be extracted, they were published in English, the intervention involved a prescribed exercise or educational programme to increase exercise participation and included an objective measurement of adherence to exercise. Articles were excluded if they were case studies, reviews, editorial opinions, testimonies, books or discussion papers. Unpublished data, published thesis and conference abstracts were also excluded.

### Quality assessment

The quality of included articles was assessed using a quality assessment tool [26] which consists of 20 criteria (Table 2). The standard of information required to meet each criterion was set a-priori. The maximum quality assessment score was 38 points (100%); based on three sub-categories: (1) the source population (11%), (2) study population characteristics (42%) and (3) methodological characteristics (47%). Each article was independently scored by two of three reviewers (LP, MTM, EC) and when agreement could not be met, the third assessor was consulted to ensure consensus was reached.

**Table 2:** Quality assessment criteria and scores used to rate the articles [26]

Category	Criteria	Scores
(1) Source population		
A	Description of source population	Not available (0) Ambiguous (1) Available (2)
B	Description of inclusion/and/or exclusion criteria	
(2) Study population characteristics		
C	Age	Not available (0) Partially available (1) Available (2)
D	Gender	
E	Education	
F	Employment status	
G	Marital status	
H	Comorbidity	
I	Economic status	
J	Data presentation of relevant O/M	
(3) Methodological characteristics		
K	Representative population	Not clear (0) Partially (1) Yes (2)
L	Study design/study type	Not clear (0) Cross sectional design (1) Retrospective/mixed design (2) Prospective design (3)
M	Population selection	Non randomised (0) Randomised/NA (1)
N	Instruments used	Non validated (0) Partially validated (1) Validated (2)
O	Statistical methods for O/M	Non appropriate (0) Partially appropriate (1) Appropriate (2)
P	Control for confounding variables	Not considered (0) Partially considered (1) Fully considered (2)
Q	Response rate versus drop outs	< 60%/not mentioned (0) 60–80% (1) > 80% (2)/NA (2)
R	Characteristics of drop outs	Not reported (0) Reported (1)/NA (1)
S	Relevant O/M	Not well defined(0) Well defined (1)
T	Limitations	Not considered (0) Partially considered (1) Fully considered (2)

### Summary measures

The following data were extracted: study design, sample population, aim of study, intervention type, length and frequency of the exercise intervention, outcome measures with time points, measures of adherence, dropout rates, rates of adherence and conclusion of the study. Where no adherence data were provided, the rate of adherence was calculated where data were available. Correlations of ≥ 0.3, ≥ 0.5 and ≥ 0.7 were considered small, moderate and large, respectively [27].

## Results

### Outcome of the search

**Fig. 1:**
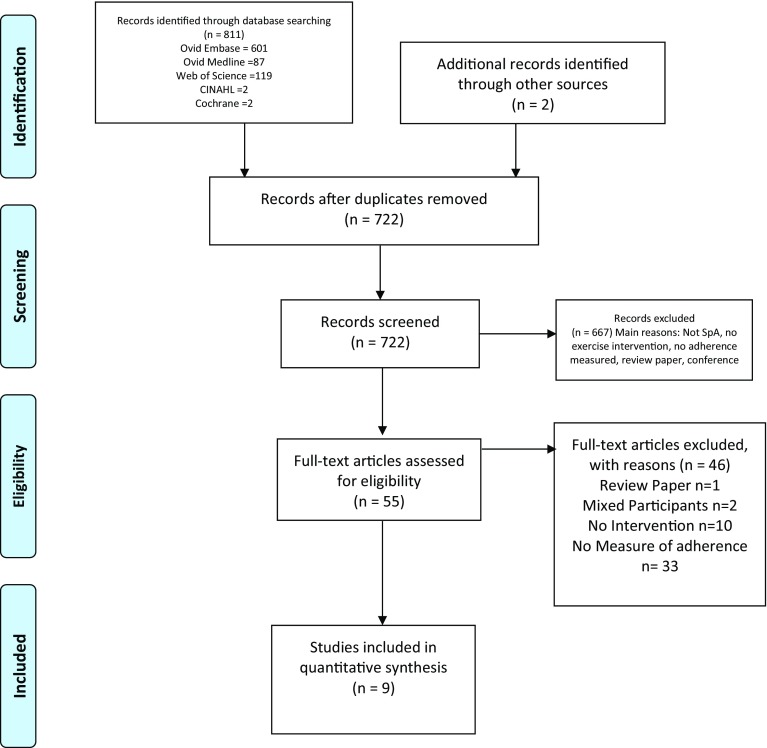
PRISMA flowchart of screening and inclusion process of included trials

The literature search produced 813 articles, including 91 duplicate articles which were removed (Fig. 1). The titles and/or abstracts of articles were screened initially by two reviewers (MTM and DM) which resulted in a further 667 being excluded. The two reviewers (MTM and DM) then examined the abstracts and full texts of the remaining 55 articles and a further 46 articles were excluded. Reasons for exclusion at each stage are provided in Fig. 1. This resulted in 9 full text articles for review and assessment. The main findings of each of the nine included studies are presented in summary tables (Table 3).

**Table 3:** Main finding from studies

Author, date, design and sample population	Aim of study	Intervention (FITT)	Outcome measures and time points	Adherence	Drop out rate	Rate of adherence and factors affecting adherence	Conclusion of study
Chimenti et al. [34] Cohort Study *N* = 30 PsA	To evaluate the effect of an exercise programme on disease activity and quality of life in PsA (minimal disease activity + anti-TNF and DMARD) patients	*N* = 30 home exercise programme for 40 min twice per week for 12 weeks	Disease Activity (VAS, tender and swollen count) Quality of Life (SF-36, global health score) Physical Activity (IPAQ) (0, 12 weeks)	Adherence was an outcome measure Patient reported exercise diaries of frequency of exercises completed	*n* = 7 (23%)	23 remaining participants completed 100% of the programme 7 participants who dropped out taken at 0% adherence Overall adherence 76.6%	Self-reported health outcomes improved in those who completed the study
Niedermann et al. [29] RCT *N* = 106 AS	To evaluate moderate intensity CV training on CV fitness and perceived disease activity in AS	Group 1 *n* = 53 Supervised Nordic walking moderate intensity (55%-85% of maximum HR) twice per week for 12 weeks and flexibility ex class 1 h per week + 1 unsupervised endurance activity such as Nordic walking or biking Group 2 *n* = 53, 3 × 2.5 h psychology led discussion on mindfulness-based stress reduction and flexibility class 1 h per week	Primary Outcome Measure; CV fitness (0, 12weeks) Secondary: BASDAI Additional outcomes: BASFI, BASMI, BASG, Physical activity, Anxiety and Depression, CRP	Adherence not an outcome measure Measured with self-monitored participant diary to supervised and unsupervised CV activities	*n* = 7 in total 7% *n* = 4 (8%) supervised Nordic walking *n* = 3 (6%) in psychology group	Adherence to CV training only reported Group 1 *n* = 40 did at least 3CV training/week (mean 3 per week) *n* = 8 not performed at least 1CV training/week *75% adherence rate Group 2 *N* = 20 mean of 1 CV /week *50% adherence rate Attended at least 2 psychology sessions: *n* = 32 Not attended at least 1 session (*n* = 10)	CV training and flexibility exercises increased fitness and reduced peripheral pain (BASDAI)
Fernandez-de-las-Penas [33] RCT *N* = 40 AS	To evaluate the long-term effect of two exercise interventions on function and mobility in AS	Group 1 *n* = 20, 15 × 1 h sessions of conventional supervised exercise programme over 4 months Group 2 *n* = 20 × 15, 1 h sessions of global posture re-education supervised over 4 months Both groups asked to continue exercise regime individually, unsupervised once per week for 1 year	Primary Outcome Spinal mobility (BASMI) Function (BASFI) Disease activity (BASDAI) (baseline, 4 months, 1 year)	Adherence not an outcome measure Adherence reported to exercising independently unsupervised for 1 year Verbally asked participants at the end-of-the-year follow-up	*n* = 0	Groups 1 and 2 80% had done exercise every week 20% did mean of 3.25 per month *95% adherence over 12 months	Global posture re-education offers short- and long-term promising results in management of AS
Hidding et al. [31] RCT *N* = 144 AS	To study the relation between disease duration and the effects of physical therapy	Supervised individual therapy of 12 supervised treatments for 30 min two times per week and encouraged to continue exercises at home for 30 min daily	Primary outcomes measures: spinal mobility, physical fitness, functioning and pain	Adherence not an outcome measure. Measured to home exercise programme only	*n* = 0	Average of 3 h doing home exercise programme *86% adherence	Short-term supervised individual therapy is effective in AS, improving mobility, fitness, functioning and global health, irrespective of disease duration
Hidding et al. [32], RCT, *N* = 144, AS patients	To study the effects of adding supervised group physical therapy to unsupervised individual therapy in AS	All participants received 6 weeks of individual supervised physiotherapy (2 × 30mins per week) and advised to do individualised HEP 30 min per day then randomised into Group 1 *n* = 68 group physiotherapy (3 h 1 h physical training, 1 h sporting activities and 1 h hydrotherapy) Group 2 *n* = 76 no group therapy	0,3,6,9 months Spinal mobility Function Physical fitness Pain and Stiffness	Adherence not as outcome measure Exercise class register of attendance for Group 1 and self-reported home exercise diaries for both groups	*N* = 9 in total 6% *n* = 1 in group physiotherapy *n* = 8 in individualised physiotherapy	Group 1 Average class attendance at class was 73.5% over 9 months Group 1 and 2 Participants median 2.6 (or 3 h average as reported in previous paper) hours on home exercise before randomisation *86% adherence During 9 months all participants spent median 1.9 h or 2.2 h average as reported in subsequent (Hidding 1994) *63% adherence No significant difference between groups	Group physiotherapy was superior to HEP in improving spinal mobility, fitness and self-reported global health
Hidding et al. [30] RCT *N* = 68 AS patients (follow-up to Hidding 1993, *n* = 68 were group 1 of the Hidding 1994 study)	To evaluate if beneficial effects with supervised group physiotherapy continued when supervised group exercise stopped	After 9 months of supervised group physiotherapy *n* = 68 people were advised to continue individualised HEP daily for 30 min and assigned to group 1 and 2 Group 1 *n* = 34 supervised group physiotherapy 3 h per week (1 h physical training, 1 h sporting activities and 1 h hydrotherapy) Group 2 *n* = 34 no group physiotherapy	Spinal mobility, physical fitness, functioning, global health (0, 3, 6, 9 months)	Exercise class register of attendance for Group 1 and self-reported exercise diaries for both groups	Overall *n* = 8(6%) Group 1 *n* = 4 Group 2 *n* = 4	Group 1 Mean 62% attendance at 3 h of supervised physiotherapy over 9 months Average over 9 months 1.8 h/week Groups 1 and 2 average of 1.8 h home exercise per week *51.4% adherence to 30 min daily HEP over 9 months Mean duration 1.9 versus 1.2 h per week for HEP for group with supervised exercises versus group with HEP only *p* < 0.05	Global health and functioning are sustained or improved if group physical therapy is continued
Sweeny et al. [28] RCT *N* = 200 AS patients	To evaluate the effect of a home-based self-care package (containing exercise)	Group 1 *n* = 100 exercise video, educational booklet, exercise progress wall chart and stickers. No information on how long participants were advised to exercise Group 2 *n* = 100 no intervention	Function (BASFI) Disease activity (BASDAI) Well-being (BAS-G) Exercise self-efficacy (Stanford self-efficacy scale) (0, 6 months)	Time of AS exercise and aerobic exercise at baseline and at 6 months	*N* = 45 in total (22%) Group 1 intervention *n* = 20 (10%) Group 2 control *n* = 25 (12%)	Group 1 Baseline; 55 min per week AS exercise, 67 min per week aerobic exercise 6 months; 99 min per week AS exercise and 85 min per week aerobic exercise Group 2 Baseline; 50 min per week for AS exercise and 72 min per week for aerobic exercise 6 months; 55 min per week each for AS and aerobic exercise Significant between group difference at 6 months for aerobic and AS specific exercise	An exercise intervention package to promote self-management significantly increases self-reported levels of exercise, self-efficacy for exercise and a trend for improvement in function
Barlow and Barefoot [35] Quasi-experimental, *N* = 52 AS patients	To examine the effect of group patient education on self-efficacy, psychological well-being and performance of home exercise	Group 1, intervention: *n* = 24 2 day self-management course, education, exercise, hydrotherapy, motivation Given a guidebook with exercise but no information on dose Group 2 *n* = 28 no intervention	Primary outcome: self-efficacy Secondary: disease severity (self-reported scale) Psychological well-being (CES-D) Physical well-being (Functional index) Home exercise activities	Adherence measured as number of home exercise activities, frequency of exercise sessions per week in the past week. (baseline, 3 weeks and 6 months)	*N* = 3 all in intervention group (11%)	Group 1 Median exercise frequency Baseline: 2.5/week 3 weeks: 6/week 6 months: 1.5/week Range Baseline: 4.5/week 3 weeks: 9/week 6 months: 7/week Group 2 Exercise frequency Baseline: 3/week 6 months: 2/week Range Baseline: 5.5/week 6 months 5.5/week Rise in range home exercise activity: baseline 3 weeks post intervention group (*p* = 0.004) and increase in frequency of home exercise sessions (*p* = 0.0023) Change in exercise range and frequency 3 weeks post intervention 6 months: decreased significance (*p* = 0.04 and *p* = 0.007) Severity positively associated with exercise range and frequency of exercise (*r* = 0.35, *p* < 0.001 and *r* = 0.28, *p* < 0.05) Longer diagnostic delay associated with performance of a great range (*r* = 0.28, *p* < 0.05) and frequency of home exercise activities (*r* = 0.27, *p* < 0.05)	Self-management course improved self-efficacy, psychological well-being at 6 months. Improvements in home exercises at 3 weeks but not maintained at 6 months
Gross and Brandt [36], quasi-experimental 18 AS patients	To evaluate if a support group helps people cope with their disease and increases their knowledge and compliance with treatment	Group 1 *n* = 11 90 min discussion per week for 4 weeks with multi-disciplinary team Group 2 *n* = 7 no intervention	Questionnaire on coping with AS, family relationships, adherence to exercise programmes and knowledge of the condition 0, 4 weeks	Questionnaire asking frequency to exercise programme the day before	No drop outs	Group 1 Attendance at ESG: mean 3 sessions *27% Compliance with exercise Improved *n* = 4, unchanged *n* = 5, deteriorated *n* = 1 No significant difference with compliance pre and post group Group 2 Compliance with exercise Improved *n* = 1 Unchanged *n* = 3 Deteriorated *n* = 2	Improvements in knowledge of disease. Compliance with prescribed exercise programmes improved but not significantly

*AS* ankylosing spondylitis, *PsA* psoriatic arthritis, *TNF* tumour necrosis factor, *DMARD* disease modifying anti-rheumatic drugs, *VAS* visual analogue scale, *IPAQ* international physical activity questionnaire, *RCT* randomised controlled trial, *CV* cardiovascular, *BASDAI* bath ankylosing spondylitis disease activity index, *BASFI* bath ankylosing spondylitis functional index, *BASMI* bath ankylosing spondylitis metrology index, *BASG* bath ankylosing spondylitis global score, *CRP* C-reactive protein, *ESG* educational support group, *HEP* home exercise programme

*indicates adherence was calculated where data was available

### Quality assessment and risk of bias

Quality assessment scores ranged from 47 to 81% (Table 4). The majority (*n* = 6) of the included articles were rated as good quality, scoring greater than 60% [28–33] (Table 4). Gross and Brandt [36] had the lowest score (47%) due to a small convenience sample (*n* = 18) and attribution bias with an average of three participants attending the weekly intervention. Two studies scored 50% [34, 35] due to poor reporting of study population characteristics. Three studies ran consecutively using the same participants [30–32]. This may have led to a repeated sample effect where a positive bias was created by the participants learning effect from the outcome measures [31–33]. In the first study, participants (*n* = 144) all received supervised exercise and a home exercise programme (HEP) for 6 weeks [32]. The participants were then randomised into two groups, an intervention group (*n* = 68) which received supervised exercise and a HEP and a control group which received only a HEP (*n* = 76) for 9 months for a second study [31]. In the third study the intervention group from the second study (*n* = 68) was divided into two groups; one group undergoing group supervised exercise and a HEP while the second group continued a HEP only for a further 9 months [30].

**Table 4:** Quality assessment tool [26] scores

Study	Source population				Study population characteristics										Methodological characteristics												Quality scores	
	A	B	To	%	C	D	E	F	G	H	I	J	To	%	K	L	M	N	O	P	Q	R	S	T	To	%	Overall total	%
Hidding et al. [30]	2	2	4	100	2	2	2	1	2	0	2	2	13	81	2	3	1	1	2	0	2	0	1	0	12	67	29	76
Barlow and Barefoot [35]	1	2	3	75	1	2	0	0	0	0	0	2	5	31	1	3	0	2	2	0	1	0	1	2	12	67	19	50
Hidding et al. [31]	2	2	4	100	2	2	2	1	2	0	2	2	13	81	2	3	1	1	2	1	2	0	1	1	14	78	31	81
Fernandez-de-las-Penas [33]	2	2	4	100	2	2	0	0	0	0	0	2	6	37	2	3	1	2	2	0	1	0	1	2	14	78	26	68
Niedermann et al. [29]	2	2	4	100	2	2	0	0	0	0	0	2	6	37	2	3	1	2	2	2	2	0	1	2	17	94	27	71
Chimenti et al. [34]	1	1	2	50	2	2	0	0	0	0	0	2	6	37	1	3	1	2	2	0	1	0	1	0	11	61	19	50
Sweeny et al. [28]	2	1	3	75	2	2	0	0	0	0	0	2	6	37	2	3	1	2	2	0	1	0	1	2	14	78	23	61
Gross and Brandt [36]	1	0	1	25	2	2	0	0	2	0	0	1	7	44	2	3	0	0	2	0	2	1	0	0	10	56	18	47
Hidding et al. [32]	2	2	4	100	2	2	2	1	2	0	2	2	13	81	2	3	0	1	2	1	0	1	1	1	12	67	29	76

### Study design and characteristics

The majority of included studies were randomised control trials (RCTs) (*n* = 5) [28–31, 33], while the remaining trials were prospective cohort studies (*n* = 2) [32, 34] and quasi-experimental studies (*n* = 2) [35, 36]. Of the five RCTs Neidermann et al. compared supervised Nordic walking and an unsupervised cardiovascular (CV) session with a discussion of mindfulness [29], Fernandez-de-las-Penas et al. compared two different types of HEP following a 12-week supervised exercise programme [33], Hidding et al. compared supervised exercise plus a HEP with a HEP only [30, 31] and Sweeny et al. compared home-based self-care programme, which consisted of an educational programme and a HEP, with no intervention [28]. Of the two prospective cohort studies; Chimenti et al. investigated a HEP only [34] and Hidding et al. supervised exercise and a HEP [32]. The quasi-experimental studies compared a self-management course with no intervention [35, 36].

### Participant characteristics

A total of 658 participants, 69% males, with a mean age of 46 years were included. Eight trials included participants with AS; 628 participants (95% of total participants) with a mean disease duration of 15 years [28–33, 35, 36], while the remaining trial included 30 participants with PsA [34].

### Measurement of adherence

Adherence to prescribed exercise was the primary outcome in four  studies [28, 34–36]. The remaining studies recorded adherence as a measure of fidelity to the exercise intervention [29–33].

Six studies measured adherence with patient-reported home exercise diaries [28–32, 34]. Four of these also reported the minutes of exercise per week, [28, 30–32]. One study asked participants to tick a box to record that the prescribed exercises had been completed [34] and a further study provided no details [29]. In the remaining three studies, participants were asked to retrospectively record their adherence at different time periods; namely, whether they had completed their exercises the previous day [36], the frequency and volume of exercises in 1 week [35], and how often the exercises had been completed over the past year [33].

### Measures of adherence and factors affecting adherence

#### Adherence to supervised exercise and a HEP

Four studies combined supervised exercise and a HEP. Nierdemann et al. reported 75% of sessions completed to three times per week supervised and a HEP over 12 weeks [29]. Hidding et al. [32] reported 86% of minutes of HEP completed within a twice weekly 30-min supervised exercise programme and a daily 30-min HEP, no additional rate was reported to the supervised sessions [32]. Hidding et al. [30, 31] reported mean adherence rates, recorded as minutes of exercise, of 63% and 51.4% for the participants receiving a HEP over 9 months. Some participants received supervised exercise in addition to a HEP; however, they did not report separate adherence rates for each group. In Hidding et al. [31] there was no difference between the groups but within Hidding et al. [30] the group with a supervised component spent significantly longer on their HEP (mean duration 1.9 versus 1.2 h per week *p* < 0.05). In addition to adherence rates for a HEP, Hidding et al. reported 74% and 62% of supervised sessions attended over 9-months [30, 31].

Three studies, Hidding et al. demonstrated that adherence to a HEP reduced over time with 86% of prescribed minutes of exercise completed in the first 6 weeks [32], reducing to 63% over the following 9 months [31], and 51% over subsequent 9-month period [30]. Adherence to once-weekly supervised exercises similarly reduced over time from 74% (attendance at sessions) in the first 9 months to 62% in the second 9-month period studied [30, 31].

#### Adherence to HEP only

Two studies measured adherence to a HEP only. Fernandez-de-las-Penas et al. [33] reported 95% adherence (sessions completed) to a once-weekly HEP for 1 year and Chimenti et al. [34] reported 100% adherence to sessions and exercises prescribed during a 12 week, twice-weekly HEP but reported 23% of participants dropped out of the programme and so calculated their overall adherence as 76%. Chimenti et al., also reported that adhering to a HEP was not affected by age, gender, body mass index, blood pressure or heart rate [34].

#### Adherence to exercise following an educational programme

Three studies measured adherence to exercise following an educational programme but did not set the dose of exercise and therefore percentage adherence could not be calculated. Barlow and Barefoot [35], found an increase in the number of completed exercises (*p* = 0.004) and frequency (*p* = 0.002) of HEP 3 weeks after a 12-h, 2-day educational programme which included information on AS, exercises in the hydrotherapy pool, posture and exercise motivation sessions. The number and frequency of exercises significantly decreased at 6 months (*p* = 0.04 and *p* = 0.007, respectively). The authors also reported a moderate but statistically significant correlation with participants with higher disease severity, having higher adherence to the number (*r* = 0.35, *p* = < 0.001) and weak but statistically significant frequency of therapeutic exercises (*r* = 0.28, *p* < 0.05), and those with longer diagnostic delay adhering to a greater number (*r* = 0.28, *p* < 0.05) and frequency of home exercise activities (*r* = 0.27, *p* < 0.05). Disease severity was measured with a similar questionnaire to the current measure of disease activity the Bath Ankylosing Spondylitis Activity Index [37].

Gross and Brant [36] reported no significant increase in exercise participation following a 4 week, once-weekly, 90-min educational session. However, they reported that four people improved their ‘compliance’ with exercise programmes, five peoples compliance was unchanged and one person had reduced compliance. While Sweeny et al. [28] found participants who received an educational video with an exercise regime, a booklet and wall chart to encourage adherence to regular exercise did significantly more “AS exercise” (*p* = 0.05) and aerobic exercise (*p* = 0.001) than a control group which received no intervention; 67 min/week of AS-specific exercise before the intervention and 99 min/week following the intervention in the intervention group, while the control group reported only an improvement of 5 min from 50 to 55 min.

### Characteristics of interventions

Exercise duration ranged from 6 weeks [32] to 16 months [29] across the nine studies. Frequency of exercise sessions varied from daily [30–32] to once-weekly [33], with individual session duration ranging from 30 min [34] to 3 h [31]. Type of interventions included hydrotherapy, Nordic walking, supervised and unsupervised, aerobic and flexibility exercises [28–36]. Educational programmes varied between 2 days to 4 weeks with individual sessions ranging from 90 min to 12 h [35, 36]. All but two studies [30, 31] used exercise interventions of varying length and frequency. There was no clear relationship between the frequency of the exercise and adherence with 95% adherence reported for a once-weekly intervention, 77% reported for twice-weekly, 75% reported for three times per week and between 51.4%–86% reported for five times per week.

## Discussion

This is the first systematic review to explore the level of and factors affecting adherence to prescribed exercise in people with SpA. Of the nine papers included, adherence rates to the exercise programmes ranged from 51 to 95%. Inclusion of education programmes and supervision, disease severity and delays in diagnosis were factors identified which may influence adherence in SpA; however, these factors were only identified in single studies, with no consensus across studies [30–33, 35]. Adherence appeared to decline over time. The exercise interventions differed in terms of frequency, type, intensity, and length and in the measurement of adherence, making direct comparison difficult.

Severity of disease and delay in diagnosis were found to influence adherence in one study, with limitations, within this review [35]. As these correlations were moderate-to-weak, they should be interpreted with caution. However, greater disease severity has been shown to be associated with better adherence in other clinical conditions [38]. It is possible that prescribed exercises could reduce disability, thus increasing motivation for people with higher disease severity, or longer diagnostic delays, to adhere to recommended exercise interventions. One small study, with limitations, within this review found completing a home exercise programme was not affected by age, gender, body mass index, blood pressure or heart rate [34]. It is likely that other personal and disease characteristics influence adherence in SpA but no further information was found in the literature within this review. Future research could investigate a variety of personal and disease characteristics that may influence adherence and consider which ones best predict adherence. Understanding who is likely to adhere to prescribed exercise can allow physiotherapists to assess who is likely to benefit from their interventions and ensure resources are put in place for those who require them.

This review found limited evidence that interventions which include supervised components and educational programmes increase adherence to exercise in SpA. Two out of three studies within this review found an increase in adherence following an educational programme incorporating exercise prescription [28, 35]. The third found only a trend towards improvement, although poor patient participation with the educational programme could account for this result [36]. Two studies within this review combined a supervised component and HEP [30, 31], one of which found that participants who were supervised for part of their programme spent significantly longer performing HEP. This review cannot conclude the magnitude of the influence of supervision and educational programmes on adherence, but it is probable that they have some effect. Indeed, supervised programmes in other patient cohorts have reported better adherence [21] and a Cochrane review of physiotherapy interventions for people with ankylosing spondylitis has shown that supervised programmes improve spinal mobility and overall wellbeing more than individualised home exercise programmes [18]. It is possible that improved adherence may in part account for this. Educational support groups have been shown to increase adherence with medicines [39].

This review found adherence to exercise in SpA declined over time following an educational and exercise programme [30, 31, 35]. This concurs with the wider field of adherence literature [15, 24, 40]. Continued adherence has been shown to depend on the ability to accommodate exercises within everyday life and the perception that exercise is effective in improving unpleasant symptoms [41]. Improving self-regulation may help to maintain adherence to exercise over time. Self-regulatory skills, a core component of social cognitive theory, could be improved through the use of goal setting, self-monitoring, self-reinforcement, stimulus control, and cognitive restructuring strategies. Previous systematic reviews in other conditions have found these strategies to be effective but as yet have not been investigated in SpA [21, 42].

Designing interventions which are underpinned by behavioural change theory such as social cognitive theory, are likely to maximise the potential for adherence to prescribed exercise and should be tested in SpA [42]. Improving health knowledge and self-efficacy are integral to initiating and maintaining behaviour change within social cognitive theory [38]. Self-efficacy refers to the magnitude of a person’s belief in their ability to undertake a task and achieve a desired goal [42]. Interventions which provide supervision and educational information at key points and/or in novel ways, such as through tele-rehabilitation, could facilitate adherence, especially in the longer term when adherence declines and warrant further investigation [43].

This review could not conclude whether the frequency of exercise sessions or the type of exercise affects adherence. Adherence to prescribed exercise may be influenced by multiple factors such as time commitment and the disease characteristics of the individual. Enjoyment and perceived benefit of types of exercise have been shown to be facilitators to regular exercise [44]. A concordance approach may improve adherence, where a physiotherapist considers how often an individual realistically thinks they can carry out their prescribed exercises, which type of exercise they would prefer and prescribes them on this basis [11]. Agreed goals and exploring barriers to change could help improve adherence on an individual basis and have been shown to improve adherence in other health conditions [45, 46]. No study within this review reported full adherence to a prescribed exercise programme. Health professionals should be aware that SpA patients are unlikely to fully adhere to an exercise programme, affecting the effectiveness of this intervention [10]. Future research should consider what level of adherence is necessary for prescribed exercise in SpA to be effective. Furthermore, there is no gold standard measure of adherence to prescribed exercise programmes. Self-reported HEP diaries, used by six of the studies within this review, may be influenced by participants’ attitudes and beliefs, poor recall, and giving a perceived desired response rather than an accurate one [47–49]. The highest rate of adherence within the included studies was 95% for a once-weekly HEP [33]. Poor recall could have influenced this rate as participants were asked about adherence after 1 year. In comparison, class attendance registers, used in all supervised components within this review, do not take into consideration the adherence to exercises within the attended exercise session [30, 31]. Developing a standardised measure of adherence, which addresses the limitations of self-reported measures and fully measures adherence, would improve the ability to meaningfully assess adherence rates and make comparisons across studies but to the best of our knowledge this does not exist.

Only 5% of patients within this review were diagnosed with PsA with the remaining participants diagnosed with AS. No studies examined adherence to exercise programmes in people with reA, uSpA or enteropathic arthritis. Therefore, the limited evidence base to date is predominantly in relation to people with AS.

This review has a number of limitations. Firstly, only papers available in English were included as there were no resources for translation. This potential publication bias may influence the generalisability of the review. It was also limited by the heterogeneity of the study designs included. Due to the variety of outcome measures used, it was not possible to conduct a meta-analysis. Three studies within this review used the same participants, this may have led to a repeated sampling bias effect which may have occurred through a learning effect of the outcome measures or a reduction in performance due to boredom.

## Conclusion

This review has found limited information on the level and factors influencing adherence in SpA. Adherence was poorly reported within included studies; however, findings suggest patients do not fully adhere. Factors identified within single studies as possible influencers were supervision, inclusion of education programmes, higher disease severity and delay in diagnosis. The full picture of adherence levels and factors affecting adherence to prescribed exercise in SpA remains unclear. Future research should aim to measure adherence to prescribed exercise over the longer term and consider multiple personal and interventional factors which potentially could influence adherence in SpA.
